# Facial Expression Recognition for Measuring Jurors’ Attention in Acoustic Jury Tests

**DOI:** 10.3390/s24072298

**Published:** 2024-04-04

**Authors:** Reza Jamali, Andrea Generosi, Josè Yuri Villafan, Maura Mengoni, Leonardo Pelagalli, Gianmarco Battista, Milena Martarelli, Paolo Chiariotti, Silvia Angela Mansi, Marco Arnesano, Paolo Castellini

**Affiliations:** 1 Department of Industrial Engineering and Mathematical Sciences, Università Politecnica delle Marche, Via Brecce Bianche 12, 60131 Ancona, Italy; r.jamali@staff.univpm.it (R.J.); j.villafan@pm.univpm.it (J.Y.V.); m.mengoni@univpm.it (M.M.); l.pelagalli@univpm.it (L.P.); m.martarelli@staff.univpm.it (M.M.); p.castellini@staff.univpm.it (P.C.); 2 Department of Engineering and Architecture, Università di Parma, Parco Area delle Scienze 181/A, 43124 Parma, Italy; gianmarco.battista@unipr.it; 3 Department of Mechanical Engineering, Politecnico di Milano, Via Privata Giuseppe La Masa, 1, 20156 Milano, Italy; paolo.chiariotti@polimi.it; 4 Università Telematica eCampus, via Isimbardi 10, 22060 Novedrate, Italy; silviaangela.mansi@uniecampus.it (S.A.M.); marco.arnesano@uniecampus.it (M.A.)

**Keywords:** affective computing, attention recognition, deep learning, facial expression recognition, jury testing

## Abstract

The perception of sound greatly impacts users’ emotional states, expectations, affective relationships with products, and purchase decisions. Consequently, assessing the perceived quality of sounds through jury testing is crucial in product design. However, the subjective nature of jurors’ responses may limit the accuracy and reliability of jury test outcomes. This research explores the utility of facial expression analysis in jury testing to enhance response reliability and mitigate subjectivity. Some quantitative indicators allow the research hypothesis to be validated, such as the correlation between jurors’ emotional responses and valence values, the accuracy of jury tests, and the disparities between jurors’ questionnaire responses and the emotions measured by FER (facial expression recognition). Specifically, analysis of attention levels during different statuses reveals a discernible decrease in attention levels, with 70 percent of jurors exhibiting reduced attention levels in the ‘distracted’ state and 62 percent in the ‘heavy-eyed’ state. On the other hand, regression analysis shows that the correlation between jurors’ valence and their choices in the jury test increases when considering the data where the jurors are attentive. The correlation highlights the potential of facial expression analysis as a reliable tool for assessing juror engagement. The findings suggest that integrating facial expression recognition can enhance the accuracy of jury testing in product design by providing a more dependable assessment of user responses and deeper insights into participants’ reactions to auditory stimuli.

## 1. Introduction

The auditory experience of a product is not just ‘a sensory response to an acoustical stimulus.’ Users attribute characteristics, such as trustworthiness or a high-quality standard, to products based on their sound production. The sounds of a product influence the users’ reasoning, their emotional state, the affective relationship they have with the product, their purchase decisions, their preferences, and even their expectations regarding the product and its performance. This is because product noise is a significant factor that contributes to brand perceptions and ultimately affects customers’ decision-making [1,2]. Therefore, customers are not only focused on a product’s functional specifications but also demand increasingly high-quality sound. The significance of sound in product design is evident in a wide range of designed artifacts, such as car interiors and engines, household appliances, and even architectural spaces [1].

In developing new products, understanding the perceived quality of sounds requires considerable testing. This experimental activity is known as ‘sound quality jury testing’ (or simply ‘jury testing’). Jury tests involve asking individuals questions about the sound quality of the products, and directly engaging with individuals who assume the role of jurors. The goal of jury testing is to understand how a group of users perceive the sounds and rate the sound quality in order to use the rating as feedback for future modifications of the investigated products. Typically, the sounds used in the tests are recordings of a product’s sound under different operating conditions or the sounds of different products in the same operating conditions. By selecting a statistically representative group of potential future customers of the product and asking them a series of questions after each sound or group of sounds, the tests establish a clear relationship between sound and perceived product quality [3,4,5,6].

The questions can be anything related to the sound quality and how humans perceive the sounds, such as how annoying or pleasant the sounds are, and which one is more related to a luxurious or robust product. The questions’ formulation depends on the test’s purpose. The answers to the questions are subjective, so every answer is considered accepted. In other words, the answers are the jurors’ opinions about the sound qualities, and there is no correct or incorrect answer. Afterward, it is up to the test moderator to take some average out of the answers, extract the answers from most jurors, and consider a rating of the sound qualities [7,8,9].

The accuracy and reliability of the jury tests’ results are indispensable. It is crucial in all products where sounds, such as car design, play a central role in users’ safety. For instance, in the automotive industry, jury tests gained much attention in designing electric vehicle engine sounds to alert pedestrians or to keep the driver’s attention high. This topic has received high interest in the scientific acoustics community to verify the objectiveness of subjective jury outputs. It is thus evident that a challenging issue in jury tests is to make the measurement as reliable, repeatable, and objective as possible and limit the subjective intervention of the moderator and jurors [10,11]. In this sense, Kim et al. employed a decision error model to improve the reliability of acoustic subjective evaluation in jury tests, particularly in the case of laser printers. They demonstrated that the chance of decision error is negatively correlated with the normalized variations between the perceived acoustic stimuli. They found that the suggested decision error model will help determine and enhance the reliability of jurors’ answers [12]. This research work, however, is placed in a different reference context concerning the one proposed, focusing on the decision errors resulting from acoustic factors.

The present study aims to investigate the possibility of using facial expression evaluation to ensure the reliability of jurors’ responses about the actual involvement expressed during the test. The research hypothesis is that incorporating expression recognition reduces the subjectivity of jurors’ responses and even the moderator’s intervention. The context of the research is automotive. More specifically, the target of the jury test is car interior noise. Facial expression recognition methods can reduce bias in jury tests since facial expressions are largely unconscious and involuntary. Therefore, facial expression recognition methods can help to measure the jurors’ emotional responses more objectively than self-reported ratings or evaluations, thus making it possible to reduce the statistical sample of jurors typically used in jury tests. Furthermore, it is essential to consider the context in which the answers are provided. In ref. [13], Özcan and Schifferstein argue that more positive affective evaluation could be facilitated either by improved sound quality (e.g., less loud or less rough) or by associations to positively laden meanings, and they provide an example of a coffee machine. The sound of the Nespresso machine could be influenced by the positive associations that the brand has created through its advertising campaigns. Therefore, the influence of the context and the participants’ expectations on their responses must be considered.

The researchers in [14] studied the complexities of identifying sounds that evoke positive reactions from customers, aiming to bridge the gap between subjective experiences and objective engineering metrics. These advancements not only facilitate a deeper understanding of auditory preferences but also highlight the significance of integrating real-life elements into sound evaluation for vehicles.

On the other hand, the researchers in [15] proposed a novel deep learning methodology to evaluate the interior noise in vehicles on mechanical and affective levels by employing small data sets to find a solution to challenges such as data scarcity, which adversely affects the performance of neural networks in predicting sound quality metrics, and the interpretation of the results. Understanding how neural networks make predictions and identifying the most influential input features for sound quality prediction is crucial for model transparency and decision-making.

However, some aspects, such as using a large and diverse sample of jurors which is representative of the customer base for the product, ensuring the testing environment is standardized and free of extraneous influences [16], and use of appropriate measures and evaluation criteria [17], can increase the objectivity of jury testing. One such set of measures consists of concordance and consistency. Concordance is the degree of agreement among different jurors’ responses to the same stimulus [18]. Consistency refers to the degree of agreement of a juror’s responses across different stimuli or evaluation sessions. It can be assessed through test–retest reliability analysis, which compares a juror’s responses to the same stimuli at different points in time [19]. Assessing these measures can identify sources of variability in jurors’ responses and control for these factors in the analysis, which can increase the reliability and validity of the results.

The investigation conducted so far has raised the main research question: is there another set of measures and objective evaluation criteria that could be used to improve the jury tests’ reliability and reduce the subjectivity of jurors’ responses?

### 1.1. Correlation between Sound and Emotions

Hu et al. studied the effects of exposing participants to different sounds on the participants’ facial expressions. They applied a deep learning approach to facial expression analysis to detect emotions accurately. Then, they compared the facial expression recognition results with the score given by participants through a questionnaire, and the comparison results showed a surprisingly good agreement. In some cases, they even found that the result of facial expression analysis worked better than the questionnaire [20]. Contrarily, Huang et al. generated facial expressions corresponding to the noise annoyance threshold [21]. They made a face-to-face social survey of 7483 participants in a city in China to provide a broad measure of noise perception by questionnaire and noise level measurement by noise analyser. Then, by free-form deformation technique, the facial expression was created according to the annoyance level calculated by the survey. Meng et al. studied the effects of sound perception in the urban environment on facial expressions. The participants were exposed to three typical urban sounds, namely, traffic noise, natural sound, and community sound. A questionnaire on the evaluation of sound perception was used to compare with the facial expression results. The results show that facial expression recognition is an effective tool for sound perception research. They found happy, sad, and surprised emotions to indicate the response to acoustic stimuli [22]. Park et al. studied the effect of different soundscape stimuli on psychophysiological well-being, i.e., heart rate, electrodermal activity, respiratory rate, and facial electromyography. Laboratory experiments were performed in virtual reality (VR) and non-VR conditions. The result showed that the rural setting helps to get superior psychophysiological recovery than the urban setting. The differences between VR and non-VR conditions were insignificant in psychophysiological recovery. However, VR conditions impacted some of the physiological responses [23].

Ozseven investigated emotion recognition by speech spectrogram image processing. They estimated the spectrogram for different speeches and detected emotions using texture analysis methods. Then, they studied the success rate experimentally by support vector machines. The result showed a better success rate than emotion recognition using only speech acoustic analysis [24]. Mauri et al. used the Implicit Association Test (IAT) and emotional facial expression analysis to evaluate user experience (UX) in website navigation. Focusing on two automotive brand websites, the research involved about 160 Italian university students who participated in a modified IAT and a website interaction task, with their emotional responses recorded. The results demonstrated the efficacy of these neuromarketing methods in UX assessment, indicating significant changes in user perceptions and emotions before and after website engagement [25]. Liu et al. used the software programme FaceReader to study the emotions stimulated in older people with dementia while listening to different sounds. They affirmed that FaceReader can correspondingly recognise different emotions changing related to different sound stimuli. They found music to be the most effective sound that can induce much more emotion than other sounds, i.e., birdsong and streams [26]. Busso et al. explored the interrelation between facial gestures and speech, highlighting that this relationship varies with the linguistic and emotional content of the message. Using an audiovisual database of an actress, they showed that facial and acoustic features are highly correlated, but differ across expressive emotional states (neutral, sadness, happiness, anger). A multilevel regression model is applied to estimate facial features from speech acoustic properties, revealing that the correlation between communicative channels is significantly influenced by emotional content [27].

### 1.2. Valence and Engagement Indicators

Literature reports several studies assessing user valence and engagement associated with listening to a soundtrack and suggesting evaluating products’ pleasantness and semantic associations, starting from [28]. Any sensory property of a product (e.g., auditory loudness, shape, flavor of food) can modulate affective experiences, with pleasantness and arousal being two key factors. For example, sharp sounds enhance the crispness of potato crisps and make them more enjoyable [29], whereas the rough sound of an epilator scares people. The researchers in [30] state that the perceived loudness of a sound primarily determines auditory (un)pleasantness, whereas the perceived sharpness of the sound mainly determines arousal. The pleasantness of noise can also be modulated by introducing highly pleasant sounds [31]. Thus, studying the pleasantness and arousal of products in jury testing is crucial, mainly to determine if these measures can be used to separate the context of the test from jurors’ prior knowledge and also to understand when jurors are losing focus during the test.

Russel and Mehrabian’s PAD model (pleasure, arousal, dominance) [28] explains how affection is physically experienced and bodily expressed and how people verbally communicate the affective attributes of objects, events, and people. Pleasure is defined in the PAD model as an indicator of how pleased a person feels and refers to a positive/negative assessment (i.e., valence or hedonic tone) of a feeling caused by a person’s current condition. Arousal indicates how intense the experienced feeling is and how this feeling stimulates a person and refers to the extent of being active in and responsive to a situation. Ref. [30] explains how pleasantness and arousal are related to sound.

Ref. [32] studied how facial expression analysis can be used to understand a person’s emotional response by employing an engagement and valence model, where valence is the intrinsic attractiveness (positive) or averseness (negative) of a situation expressed by the person and is associated with the emotion being expressed [33]. Engagement is a weighted average of the discrete set of emotions (anger, disgust, fear, happiness, sadness, surprise) identified by Ekman. It indicates the active involvement of a person in the situation. Thus, valence corresponds to pleasure in the PAD model, and engagement corresponds to arousal.

These results suggest that studying the emotional connection in user-product interaction is important and that valence and engagement are two of its main components. Those two metrics can be studied via observational methods such as facial expression recognition [32].

### 1.3. Technologies for Valence and Engagement Evaluation

For a long time, the scientific community has studied the possibilities of giving an objective and quantifiable representation of a person’s level of involvement, especially analysing his/her emotional factors. Likewise, methods that classify and objectively categorise human emotions based on external signals, such as facial expressions, voice, and physiological signals, that people use consciously or unconsciously to express their emotions have been investigated. Among the best-known of these researchers, we undoubtedly have Paul Ekman, who in 1971 theorised the presence of a discrete set of distinct emotions: anger, disgust, fear, happiness, sadness, surprise, and their corresponding universal facial expressions (hence the attribution of the name ‘Ekman’s Universal Facial Expression’), expressed in the same way all over the world, beyond a human being’s ethnicity or geographical origin [34].

Research studies have suggested that analysing facial expressions could be a valuable tool in evaluating the emotional impact of sounds on people, even outside the context of jury testing [35].

Facial expression analysis aims to recognise patterns of facial expressions and link them to emotions based on a specific theoretical model. The most widely used theoretical model is the face action coding system model (FACS) [36], which allows for the identification of six universal emotions (i.e., joy, surprise, sadness, anger, fear, and disgust) by tracking the movements of the face muscles. Consequently, it is unsurprising that most algorithms developed so far allow only these emotions to be recognised.

These technologies were successfully employed in the computation of valence and engagement in [32] to evaluate metrics for satisfaction and productivity during usability tests for web platforms.

Up to now, most of the available facial expression recognition (FER) systems make use of deep neural networks [37], particularly convolutional neural networks (CNN), as in [38], which take images of human faces as input and provide a prediction of the relevant Ekman main emotions (i.e., happiness, surprise, sadness, anger, disgust, and fear) [39].

The system discussed in [40] integrated convolutional neural networks (CNNs) and support vector machines (SVMs) to perform emotion classification and attention detection. The CNN was used for facial landmark-based emotion classification, while pretrained SVM models classified faces into emotion categories and attention levels based on the calculated landmarks.

The toolkit discussed in [41] offers several key features for automatic analysis of human behavior in HCI applications in the wild. However, some limitations of this tool include the need for high-quality images for accurate facial expression recognition, potential challenges in system calibrations for gaze tracking accuracy, and the reliance on deep learning algorithms which may require significant computational resources.

Specifically, the research challenges in the field of face reader technology and emotional metrics can be broadly categorized into technical and methodological challenges.

Firstly, the accuracy and reliability of facial expression recognition (FER) systems are hindered by various factors such as illumination conditions and occlusions of face parts. To combat these limitations, employing multiple observation channels and improving illumination conditions are suggested [42]. Secondly, context interpretation is another important aspect of understanding human emotions, and this can be a difficult task to accomplish for artificial intelligence-based systems [43]. Recognizing micro-expressions, which are brief and often involuntary facial movements, presents a significant challenge as they require precise motion tracking and recognition algorithms [44,45]. Micro-expressions can also constitute a genuine preamble to certain actions [46]. For instance, they can appear during an interrogation indicating tense areas inside the psyche or they can be visible in stressful situations. Thirdly, cultural and individual variability further complicate emotion recognition, as highlighted by studies emphasizing the profound differences in emotion recognition across diverse populations [47,48,49]. The variability in how individuals express emotions underscores the complexity of interpreting facial expressions accurately and necessitates personalized approaches in emotion recognition systems.

Moreover, alternative approaches to emotion recognition involve analyzing electroencephalography signals (EEG) with machine learning models. While these methods yield competitive accuracy, a significant challenge lies in dataset creation due to constraints associated with EEG recorders and human resources [50,51,52].

Methodological challenges involve the use of non-invasive measurement tools for real-life acquisitions. It is known that the best way to recognise human emotions in real-life environments is to process video and images captured by a camera without invasive technologies such as wearable devices (e.g., helmets, bracelets) or distributed sensors. In fact, despite being generally more accurate, the use of biofeedback tools, e.g., electrocardiography (ECG) and galvanic skin response (GSR), are not suitable for real-life acquisitions [53,54]. These reasons have led the scientific community to improve the accuracy of non-invasive emotion recognition systems based on speech analysis and facial coding in recent years. Among the various studies that have gone in this direction, [55] introduces a robust multi-depth network for accurately recognizing facial expressions by leveraging a multi-depth network and a multirate-based 3D CNN, demonstrating its efficacy in understanding human emotions through facial expressions, while ref. [56] utilizes self-supervised learning models, including universal speech representations with speaker-aware pre-training, to evaluate different model sizes across sentiment and emotion tasks.

In exploring advancements in facial expression recognition (FER), the work of [57] presents a notable development through a hierarchical attention network. Their approach innovates by employing an attention mechanism that not only enhances expression-relevant information but also suppresses extraneous data. Their method aggregates diverse features through specialized feature aggregation blocks, leveraging both local and global context. Furthermore, a hierarchical attention module (HAM) systematically enhances discriminative features while filtering out irrelevant facial features. Their experiments showcase that this methodology outperforms existing FER systems, setting a new benchmark for the field.

Among the various emotion recognition methods, those based on speech analysis or facial expressions are the least invasive. However, the effectiveness of systems based only on speech-emotion recognition still needs to improve compared to systems based on facial expression recognition [58].

In addressing the challenge of attention recognition, facial emotion detection has been employed to forecast human attention allocation within visual saliency prediction scenarios, yielding favorable outcomes [59,60]. The current attention recognition systems, which are crucial for ensuring the reliability of emotional data, incorporate fuzzy logic to capture the nuances of a user’s attention level [61].

Another challenge is dataset specificity. The system described in [62] leverages innovative CNN models trained on merged datasets to improve accuracy in recognizing Ekman’s universal emotions from human faces captured in real-world scenarios. The tool combines lab-generated datasets with ‘in the wild’ datasets to create a more robust model capable of handling diverse environments. However, some limitations of this tool include accurately labeling datasets collected from the web, which may impact the model’s performance in recognizing emotions from images with inaccurate labels.

Developing accurate and reliable emotion recognition systems is a challenging process. Furthermore, in the case of jury tests, emotion recognition appears even more difficult. One of its challenges relates to the monotony of the test itself and the little attention jurors might end up placing during test execution if they are not totally committed to the test. Usually, jury testing is performed for a specific product, so related sounds are often alike and monotonous. It is challenging for jurors to decide how to answer the questions because they generally find discriminating among them difficult. Commitment and focus capabilities of the jurors are therefore key elements to a successful jury test; otherwise, the test itself can be biased and unreliable, as noted in [30]. Often, the subject can only realise the actual level of liking of sounds if they specifically arouse certain well-marked feelings related to the person’s memories or preferences (e.g., the roar of a sports car engine for a luxury car enthusiast).

The use of FER technologies, coupled with other physiological measures if available, can help provide more objective data on jurors’ emotional and cognitive responses to the products or sounds in the case of jury tests being evaluated.

### 1.4. Research Aim

In the landscape of emotional response analysis to sound stimuli, significant strides have been made, particularly through the employment of facial expression recognition (FER) systems. Despite these advancements, a comprehensive review of the literature reveals several critical gaps in the application and scope of existing studies. These deficiencies are outlined as follows:

1. Lack of application in jury testing: Previous research has extensively explored the utilization of FER systems in various contexts but conspicuously lacks application within the framework of jury testing. The innovative use of FER systems to gauge emotional responses in a jury testing scenario remains unexplored, representing a significant gap in the current body of knowledge.
2. Overlooking vehicle sound quality assessment: Another notable omission is the application of FER systems in the assessment of vehicle sound quality. Given the importance of sound quality in the automotive industry, especially concerning customer satisfaction and perception, the absence of studies leveraging FER technology for this purpose indicates a missed opportunity for a more nuanced analysis.
3. Neglecting juror involvement levels: The current literature does not adequately address the potential impact of juror involvement levels on the outcomes of jury testing. Understanding and quantifying the level of emotional engagement or detachment of jurors can provide deeper insights into the reliability and validity of jury testing results, yet this aspect remains under-investigated.
4. Failure to identify unfocused jurors: Equally important is the identification of jurors who may not be fully concentrating during the testing process. The capability of FER systems to detect subtle facial expressions indicative of distraction or lack of engagement has not been utilized in the context of jury testing. This oversight undermines the accuracy and efficacy of the results, as unfocused jurors can significantly skew the data.

By addressing these gaps, this study significantly advances the field, offering more robust and nuanced insights into people’s emotional responses to sound stimuli in jury testing. Implementing FER systems in these unexplored areas could not only enhance the methodological approach to jury testing and vehicle sound quality assessment but also ensure more reliable and valid outcomes by considering juror involvement and focus levels.

Building upon the understanding that jurors’ facial expressions can illuminate their levels of involvement and concentration, this approach offers a subtle method to enhance the accuracy of jury testing outcomes by employing facial expression recognition (FER) technology throughout the jury test, so that it becomes possible to monitor and predict jurors’ engagement in real-time. This is valuable in the jury testing process, as the level of juror involvement directly impacts the reliability of test results. Identifying moments of reduced concentration allows for the segregation of data into more and less reliable categories based on the jurors’ engagement levels. Consequently, this method leads to the possibility of filtering jury test data, ensuring that conclusions drawn are grounded in the portions of the test where jurors’ attention was most acute. Recognizing and adjusting for fluctuations in jurors’ focus not only refines the validity of the testing process but also offers insights into optimizing test design to maintain or regain juror engagement. This approach underscores the importance of integrating advanced technologies like FER into jury testing frameworks to capture the depth and variability of human responses, leading to more dependable and insightful outcomes.

The present research aims to enhance the current jury testing methodology by introducing a new facial expression analysis component that leverages the calculation of metrics, such as attention, valence, and engagement, to recognise which responses are reliable and consistent during jury testing sessions. The goal is to introduce objectivity to the responses and to establish a method to support sound quality evaluation that is replicable and easily applicable by adding a FER system. The effective potential of using automatic facial expression recognition systems to evaluate the level of user involvement during jury tests has yet to be investigated, and so have the correlations between the feedback’s reliability and the participants’ attention. This research adopts a case study where valence and engagement have been used as objective parameters to evaluate sound quality and investigate their role in jury test reliability assessment. The facial recognition system allows both to be measured. Attention is also taken into consideration during the computation of valence and engagement to investigate the correlations with participants’ answers’ reliability and provide an objective measure of it. A further purpose emerged during the investigation regarding the support that these tools can provide to analysts in understanding the reliability of subjective sound quality outcomes during jury tests.

Analysis and discussion of the results allow the research to highlight the limitations found by the application and the potential and future developments for sound design.

## 2. Materials

The application of deep learning-based software and models trained for facial expression recognition are among the most promising methodologies to evaluate valence and engagement parameters [32,63]. In recent years, the study of facial expressions has garnered significant attention across various research domains, signaling a paradigm shift in the methods used for understanding and interpreting human emotions. Traditionally, the evaluation of emotional states relied heavily on verbal cues and self-reported measures, which, while valuable, present limitations in terms of accuracy and objectivity [64]. The wide spreading of machine learning (ML) technologies has ushered in a new era where automated systems are increasingly taking the forefront in analysing facial expressions, offering a more nuanced and precise window into human affect [65].

The analysis of facial parameters provides an objective method of assessing the level of appreciation expressed by users [66], where techniques requiring direct feedback to users introduce considerable subjectivity and bias. Therefore, for this research, two software programs based on deep learning and computer vision models are proposed to extrapolate indicators of the parameters of valence and engagement that are manifested by the users during the auditory stimulation in the case study.

### 2.1. A Facial Expression Recognition Tool for Valence and Engagement Evaluation

A tool based on a CNN implemented in Python using the Keras and TensorFlow frameworks has been adopted for this particular research [62]. The current task of this network is to recognise Ekman’s six universal emotions (i.e., happiness, surprise, sadness, anger, fear, and disgust) from images of faces as input and output a percentage probability for each emotion. As described in [37], two types of datasets are generally used to train deep neural networks for facial expression recognition: datasets created in a laboratory with high accuracy and datasets collected ‘in the wild’ with lower accuracy. The CNN developed for the expression recognition software aims to combine the strengths of both datasets to create a new merged dataset. The public datasets CK+ [67], FER+ [68], and AffectNet [69] were used for this purpose as references, pre-processed to have consistent characteristics for all photos in the new dataset, and cleaned of any photos not suitable for training.

In the last layer of the proposed CNN, a softmax function returns Ekman’s emotion scores, predicted from every camera video frame, normalised to a percentage value of 100. Following the method derived from Russel’s classification of Ekman’s emotion in his valence/arousal (Circumplex) model [33], and tested in [32], valence and engagement are calculated from the percentages predicted for each Ekman’s emotion. As a result, valence ranges from −100 to 100, indicating the total positivity or negativity expressed by the participants, while engagement ranges from 0 to 100, giving a measure of how far the expressiveness of the face deviates from neutrality.

Valence and engagement are determined using Formulas (1) and (2):

Valence = Happiness(%) − Sadness(%) − Anger(%) − Fear(%)− Disgust(%).(1)

Engagement = Happiness(%) + Surprise(%) + Anger(%) + Fear(%) − Sadness(%).(2)

### 2.2. A Proposed Attention Recognition Tool to Improve Jury Test Reliability

The study exploits the facial expression recognition system and software capable of detecting whether the user is attentive or not during the test, using a combination of parameters that concern the level of rotation of the head to the camera mounted on top of the screen, the direction of gaze and percentage of eyelid opening to improve the reliability of the valence and engagement evaluation. The assumption is that where the juror has shown to be inattentive, the emotional data extrapolated from the facial expression recognition CNN for that particular instant could be less reliable than the others, mainly because the emotion resulting from the facial expressions could be driven by events or memories not relevant to the current test.

**Figure 1:**
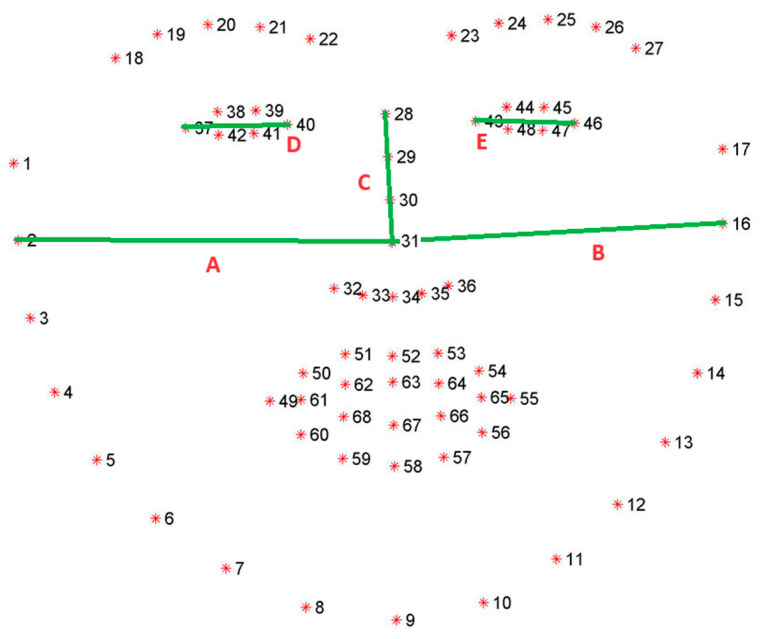
Dlib facial landmarks [70] used for attention evaluation. Green lines indicate the distances between the pairs of points used to estimate head orientation.

The third-party library Dlib has been used to retrieve a mapping of the user’s facial characteristics and, hence, to evaluate the attention level. Figure 1 shows the map of the 68 landmarks detected through the Dlib face landmark predictor model.

In particular, the distances between five pairs of points (i.e., 2–31, 16–31, 28–31, 37–40, 43–46) were considered to estimate the head orientation to the screen (Figure 1). Based on the usual symmetric characteristic of a face, such distances should remain constant between the left and right sides. Consequently, the division ratio between the distances constructed in the left part of the face and those in the right part should remain in the neighbourhood of 1. Following this approach, an estimate of the head rotation values defined as yaw and pitch values was provided, i.e., movement around the vertical and horizontal axes to a hypothetical position in front of the camera. Yaw was estimated as the absolute value ratio between distances A and B. At the same time, pitch was the ratio between distance D (or distance E if D cannot be obtained) and distance C in absolute value. The thresholds for determining whether yaw and pitch values indicate a lack of attention were determined empirically from a sample of 54 users with monitors in different configurations (different camera positions and different sizes), by assessing the yaw and pitch values at which users turned their faces to the screen. Used thresholds are defined in Algorithm 1:
**Algorithm 1**1. Result: Face attention binary value, ear value 2. Inputs: Facial landmarks X-Y coordinates 3. horizontal_left_distance = Euclidean distance between landmarks 2 and 31 4. horizontal_right_distance = Euclidean distance between landmarks 31 and 16 5. yaw = absolute value of the division between horizontal_left_distance and horizontal_right_distance 6. If yaw is greater than 1, then yaw = the reciprocal of yaw 7. yaw_attention_threshold = 0.4 8. If yaw is less than yaw_attention_threshold, face_attention = 0, otherwise face_attention = 1 9. horizontal_distance_pitch = Euclidean distance between landmarks 28 and 31 10. vertical_distance_pitch = Euclidean distance between landmarks 37 and 40 (if not null) or 43 and 46 11. pitch = absolute value of the division between horizontal_distance_pitch and vertical_distance_pitch 12. pitch_attention_threshold = 5 13. If pitch is less than pitch_attention_threshold, face_attention = 1, otherwise face_attention = 0 14. ear_attention_threshold = 0.10 15. If ear is less than ear_attention_threshold AND pitch is greater than pitch_attention_threshold, face_attention = 0

Moreover, the study adds further control to prevent users from looking downward during the jury test.

For this reason, a measure called eye aspect ratio (EAR) is considered and computed according to the method to detect eye blinking proposed in [71]. Although this method was proposed to detect eye closure, it has also been considered helpful for detecting users’ frontal head tilting. A significant decrease in the value of this ratio was empirically observed during the test phase in people looking downwards and away from the camera. To avoid false positives derived from the analysis of camera frames recorded while the user is blinking, this feature was combined with pitch evaluation. The user is considered distracted if both the EAR and Pitch are under certain thresholds, defined as indicated above. 

The attention tool integrates a gaze tracker [41] to predict the direction the user is looking. The gaze detection is performed by a CNN whose architecture is similar to the one proposed in [72]. The CNN for gaze tracking was implemented and trained using the Python programming language, running Keras API upon TensorFlow library support and with GPU acceleration.

To predict the gaze position on the screen, the CNN needs the face image (sized 224 × 224), and the two eyes images (sized 224 × 224) cropped using the Dlib [70] frontal face detector and 68 landmarks predictor, respectively, and also a 1 × 4 face grid vector, expressing the portion of the entire image occupied by the face. The output is a two-dimensional vector containing the X and Y coordinates of the estimated display point (in centimeters), referring to the top left corner as the origin of the axes. This CNN has been tested for a web-based HCI analysis tool (version 1.1) [32]. In this study, the network predictions are only used in relation to empirically defined thresholds to assess whether the user is maintaining a direction toward the monitor or not. The poor accuracy shown by the model in deriving the Y-axis gaze coordinates was not considered influential for this case study, as only the X-axis gaze direction was taken into account. This information is then added to the previous algorithm:

1. Result: Attention binary value
2. Inputs: Face attention value, gaze x-y coordinates
3. If gaze_x_coordinate <25 and gaze_x_coordinate > 1 and face_attention = 1 than attention = 1

The attention level values could be used to filter the data, providing evidence that the tester is not paying attention in the corresponding frame, avoiding analysing emotional data probably elicited by events, distractions, or thoughts not related to the current test.

The system was tested on 20 participants, comparing the real and predicted coordinates.

## 3. Methods

**Figure 2:**
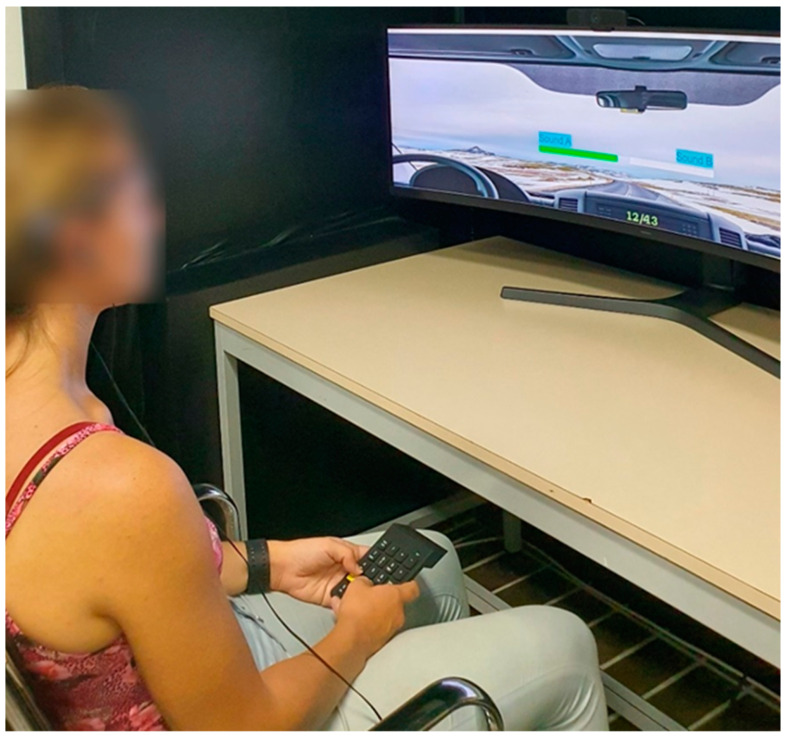
View of the test environment: - the monitor displaying a typical driver scenario and the two sound presented in the AB test and – the keyboard for the option choice. For the reading of the writing on the monitor refer to Figure 3.

The experiment was conducted at the Polytechnic University of Marche, involving 40 participants, predominantly students and university employees across various age groups, with an almost one-to-one male–female proportion (21 females, 19 males). The average age of the participants was 28 years (SD 6). During the test, a webcam, mounted on the top of the monitor, recorded every face without affecting the perception of different auditory stimuli. The following tools were used: the Windows Camera app to store the video stream taken by a Logitech HD C925E webcam connected to a PC with a recording frequency of 30 Hz at 1080 p, a software developed in Matlab for the management of the audio stimuli and for times synchronisation with the recording software, and finally, the software developed in Python v3.7 described in paragraph 3 to analyse the recorded videos. Analysis of the recorded videos involved processing 14 frames per second with the Python algorithm, yielding probabilities for each of Ekman’s expressions for each frame. The data, including probability percentages, timestamp, and attention values (Boolean true/false values) were finally stored in CSV format. A desktop PC with Intel i9 10900 CPU, Nvidia Quadro P2200 video card, and 128 GB of RAM was adopted for the image processing step. An environment designed to capture the jurors’ attention and focus on the content presented on the display has been prepared for this test. In particular, it was carefully designed to ensure participants’ attention and focus, with a dimly lit setting, reduced ambient noise through headphones, and an enclosed space free from external stimuli or sources of distraction. To maximise the immersiveness of the subject, a 49-inch Samsung Odyssey G9 curved screen was used to display a graphic interface developed in Matlab for the simulation of a driving scenario. Participants, upon arrival, were familiarized to the testing environment and provided clear instructions regarding the test protocols and their roles. This also included reading and signing an informed consent form, ensuring that all participants were well informed, willing to partake in the experiment voluntarily, and fully aware of what they agreed to, including potential risks and benefits. Figure 2 depicts the test environment.

The experiment aimed to determine whether a facial expression recognition tool can be used in the context of a jury test to enhance the reliability of the test. The jury test performed was designed according to an AB comparison approach augmented by AB–BA repetition and was intended to analyse the jurors’ reaction to seven different interior car noises, a technique already implemented in [19]. The sounds utilised in this study encompass those emitted by two different diesel engines operating in stationary mode, a sports racing car during a race, an electric vehicle in run-up operation, and three standard petrol vehicles in motion on roads. Even though this can be considered less demanding from an acoustic jury test point of view, given the marked differences in the noises involved, the validity of the test still holds. Moreover, the final goal was more related to understanding whether the FER approach can be used to increase the accuracy associated with a standard jury test rather than performing a selection of sounds based on a subjective jury test.

All seven sounds were systematically compared and repeated against each other, resulting in a comprehensive test sequence consisting of 42 sound pair comparisons. The sounds were taken from different car engines, some of which were chosen to elicit responses on an expressive level (in particular with old engines’ annoying noises). Two primary questions guided the sound comparison: ‘Which sound is more annoying?’ and ‘Which one appears to be from a higher-quality car?’. The participants were asked to answer both questions after each pairwise comparison. This approach allows the study to uncover insightful data regarding the subjective acoustic quality of different car interiors. A primary trial part was incorporated at the beginning of the test to familiarise the jurors with the test mechanics and expectations. This trial segment comprised only one sequence, including a single sound pair comparison. This initial step aimed to introduce the jurors to the test environment and process, ensuring they clearly understood the test itself. Including this preliminary trial sequence was crucial in promoting a smooth transition for jurors into the primary test.

**Figure 3:**
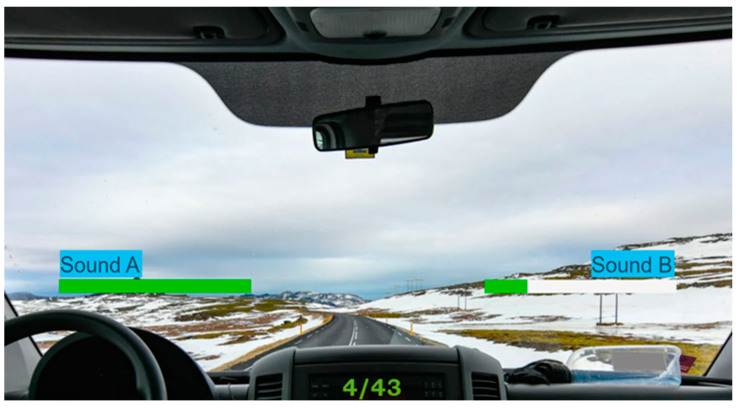
A screenshot of the graphical user interface to interact with the jurors.

Furthermore, to ensure a seamless and focused interaction with the experiment, jurors engaged with a user-friendly interface developed in Matlab. The interface application was developed using the Matlab App Designer platform to facilitate the jurors’ experimenting, offering graphical instructions and an interactive platform. The user interface design was explicitly crafted to enhance the jurors’ comfort and concentration during the experiment. Jurors were advised to maintain a relaxed demeanour, minimising extraneous movements, and focus on the acoustic test. During the experiment, the app automated the presentation of sound pairs, termed A and B, to the jurors (Figure 3). The app managed and collected the jurors’ responses to comparison questions with an external Bluetooth mini keyboard. The decision to use this device stemmed from its convenience advantages. Jurors could easily hold the keyboard in their hands, allowing for a more natural and relaxed posture during the experiment. This setup minimised eye or hand movements, enabling jurors to focus intently on the sounds and respond effortlessly by pressing the keyboard buttons corresponding to their choices. Preventing eye and head movements strengthens the accuracy of facial expression analysis, which could be spoiled by fast movements.

After the jurors finished the test, they were directed to a questionnaire accessed via a QR code. This questionnaire was designed to understand when the jurors were actually focused and involved during the experiment. It served as a tool for them to reflect and provide feedback about the test.

The questionnaire contained questions about demographic information and specific questions addressing participants’ attention. The jurors were asked for some information regarding their state of mindfulness at the beginning of the test, during the test, and at the end of the test. In particular, the jurors were asked to express their concentration levels during the different test stages. The responses to these questions could be selected on a scale ranging from ‘fully focused’ to ‘feeling mentally fatigued’, adding further insight into participants’ awareness and focus during the experiment. The provided scale included the following options:

- Fully focused
- Actively involved
- Partially focused
- Distracted
- Feeling bored
- Heavy-eyed
- Feeling mentally fatigued.

## 4. Results

**Figure 4:**
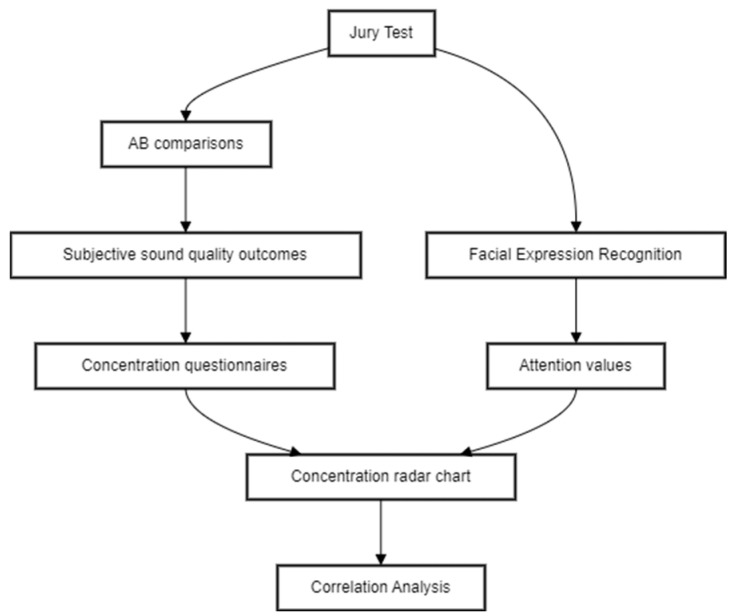
Flowchart describing the implementation steps drawn using Mermaid code.

The data analysis process comprised several steps, as shown in Figure 4: following the jurors’ participation in the test via the AB comparison approach, an analysis of their responses to the questionnaires was conducted. Subsequently, a correlation analysis between the time series data of valence and the jurors’ responses to the AB comparisons was performed, taking into account attention data from the facial recognition tool to distinguish between objective responses to the jury test.

**Figure 5:**
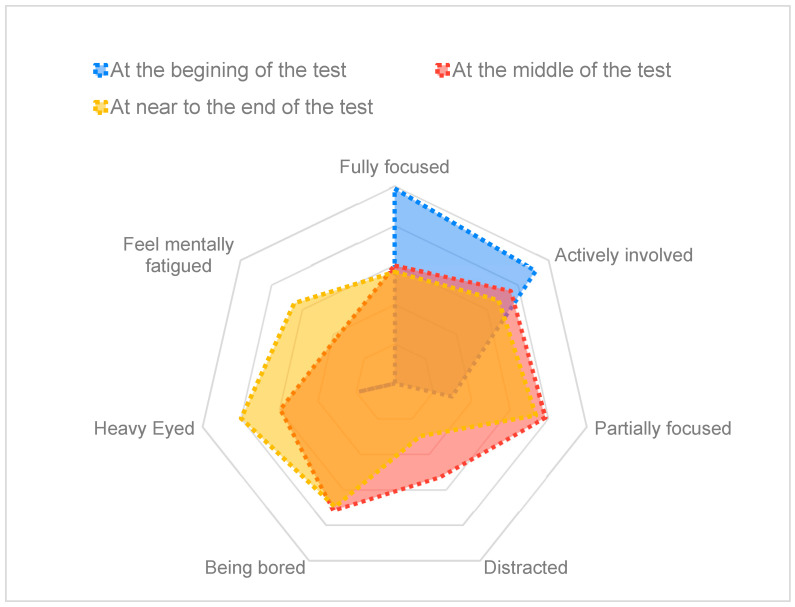
Radar chart to represent the jurors’ concentration levels throughout the test campaign.

The analysis of the questionnaires answered by the jurors was used to enhance our understanding of the jurors’ cognitive state throughout the experiment and, at the same time, to obtain feedback about the reliability of the results reported by the attention recognition tool. These results, depicted in Figure 5, revealed a trend in jurors’ concentration levels throughout the test. Initially, jurors mainly reported being ‘fully focused’ or ‘actively involved.’ However, as the test progressed, a shift was observed, with jurors indicating lower concentration levels, feeling ‘partially focused’ or ‘bored’. Especially after the middle of the test, towards the end, jurors predominantly expressed feelings of being ‘heavy-eyed’ and ‘mentally fatigued,’ meaning a decreased overall alertness and focus. This trend suggests that jurors started the test with a high level of freshness and active involvement but experienced a gradual decline in concentration, attributed partly to the lengthy duration of the test. The analysis of the questionnaires is complemented by the concurrent analysis of attention time series data. This dual approach yielded similar results, confirming that jurors exhibited reduced attentiveness toward the end of the test.

Understanding jurors’ concentration levels is pivotal in accurately interpreting a jury test’s results. The observed trends could influence the jurors’ perception and evaluation of the sound qualities, making this an essential aspect to consider when analysing and drawing conclusions from the collected data.

The attention recognition tool computes the jurors’ attention levels, expressed as binary values, as an original computation of head rotation and gaze direction estimation. The attention prediction values were extracted and arranged according to the periods reported in the questionnaires.

In this way, by correlating the tool’s calculated values with the questionnaires’ responses, we were able to evaluate the emotional values associated with different mental/concentration states from a statistical perspective.

The predicted attention, in fact, has been considered a representative metric of jurors’ concentration levels and involvement in the experiment.

**Figure 6:**
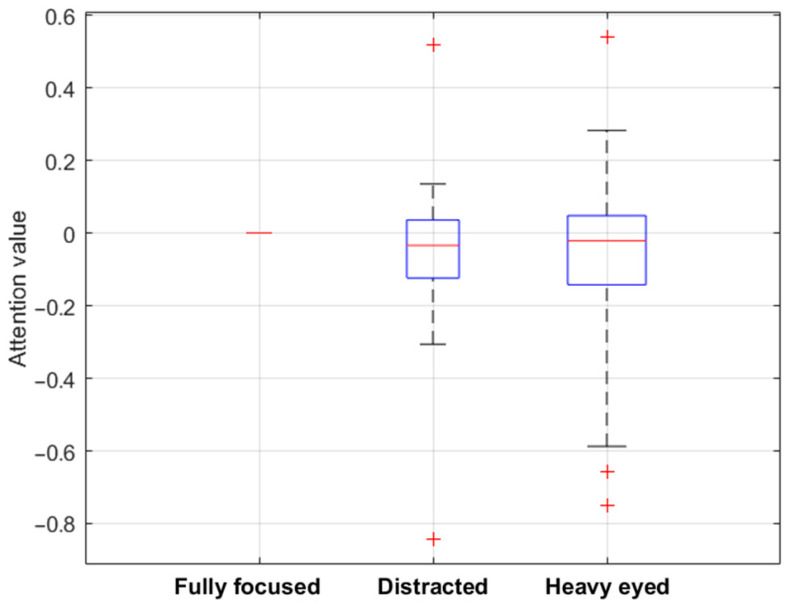
Comparative boxplot charts depicting median attention values (red lines) across various concentration statuses, with ‘fully concentrated’ status as the baseline (red line). The blue box limits represent the 25th and 75th percentile and the black whiskers indicate the extreme values not considered outliers. The red crosses stand for the outliers.

In Figure 6, the binary values obtained from the attention recognition tool were examined in conjunction with the questionnaire feedback provided by jurors, focusing specifically on three distinct concentration statuses: fully focused, distracted, and heavy-eyed. These three statuses were selected for analysis as they were the most frequently reported states of mindfulness in the jurors’ responses to the questionnaire. By evaluating these specific states, the study aims to enhance the understanding of the dependability of jurors’ responses.

Specifically, in Figure 6 the Y-axis represents the values from the tool, while the X-axis displays the categories of questionnaire responses. The figure reports the median attention values calculated across all users who responded in the three categories and across the three different periods considered in the test (beginning, during, and near the end of the test). In this way, each box plot symbolises the range of attention values associated with a specific state of mindfulness, visually representing the jurors’ attention variations corresponding to their self-reported mental states. The differences become more apparent by subtracting the attention values associated with a ‘fully focused’ status from all other attention values linked to each concentration status and setting the ‘fully concentrated’ status as a baseline with fixed zero values.

Accordingly, positive values in Figure 6 indicate increased attention levels when transitioning from a ‘fully concentrated’ status to another, while negative values signify a decrease. An observable shift below zero in the box plots related to the ‘distracted’ and ‘heavy-eyed’ statuses suggests a reduction in attention levels compared to the ‘fully concentrated’ status. It reveals an overall discernible decrease in attention levels, calculated from facial expression analysis when jurors felt distracted or heavy-eyed compared to when they were fully concentrated. Specifically, about the ‘distracted’ status, it was observed that 70 percent of jurors exhibited a reduction in attention levels as calculated by the tool. Similarly, for the ‘heavy-eyed’ state, 62 percent of jurors demonstrated a decrease in attentiveness. These insights are particularly valuable for jury test assessments, serving a dual purpose. Firstly, they contribute to a more comprehensive understanding of the overall reliability of jurors’ responses in the entire test. Secondly, they allow for an individual assessment of each juror’s reliability. This facilitates a nuanced approach where more reliable responses can be assigned greater weight in future studies and applications of sound-quality jury testing. Such differentiation not only enhances the overall accuracy of these assessments but also fine-tunes the process for more effective results.

**Figure 7:**
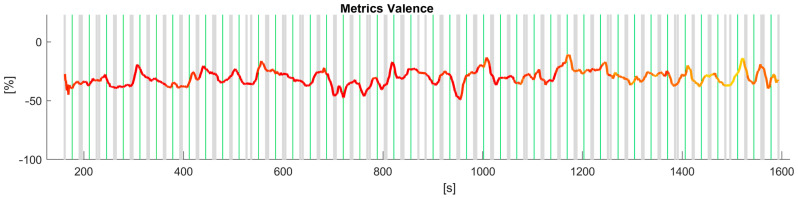
Time series data for valence indicating a decline in jurors attentiveness throughout the jury test as shown by the shift from red (attentive) to yellow (less attentive). Grey vertical bars separate each pair of audio signals, green vertical lines separate the two signals in each pair.

Then we proceeded to analyse the time series data for valence across the jurors. The preprocessing steps involved cleaning the data to ensure the quality of the analysis. Firstly, frames where no data were collected from the expression recognition tool were removed from the dataset. Additionally, frames containing null values or otherwise unusable data from the same tool were excluded. In total, they accounted for a small percentage (less than 5%) of the usable data. Furthermore, frames captured at the beginning and end of the test, when jurors were either sitting down or standing up and their facial expressions were not pertinent to the test, were also eliminated. Lastly, since attention and emotions data were both outputs of the same analysis, we did not need to employ data fusion techniques. Analysis of the time series data for valence across the jurors revealed a consistent decline in attentiveness levels as the tests advanced, in agreement with the results reported from the analysis of the questionnaires. This decline in attentiveness was prominently indicated by the shift from red, representing attentive states, to yellow, indicative of reduced attentiveness, as depicted in Figure 7. The valence data used in these and the following figures were collected through video recordings captured at a frame rate of 14 frames per second (FPS) during the entirety of the jury testing process. The data was smoothed via a moving average approach to represent the valence signals in Figure 7. Moreover, the attention signal was embedded in the valence graph. For representation purposes, the attention data was averaged as well with the same time window, yielding a range of values in the interval [0,1] extremes included. The colour was chosen based on several thresholds, when the attention is above 0.8 the valence line is red, when the attention value is below 0.2, the line is yellow, otherwise, the line is orange. An attention value equal to 1 means the juror was attentive or looking straight at the camera/monitor, whereas an attention value equal to 0 means the juror was not attentive. Thus, when the juror’s attention diminishes, its valence is represented by the colour yellow. The grey vertical bars separate each pair of audio signals administered in the AB comparison with the AB–BA repetition approach. The green vertical lines separate the two signals in every pairwise comparison. These visual representations provide clear evidence of the diminishing attention spans of some jurors for the trial, thereby emphasising the significance of utilising facial expression recognition (FER) techniques in understanding and assessing the dynamics of jury testing.

**Figure 8:**
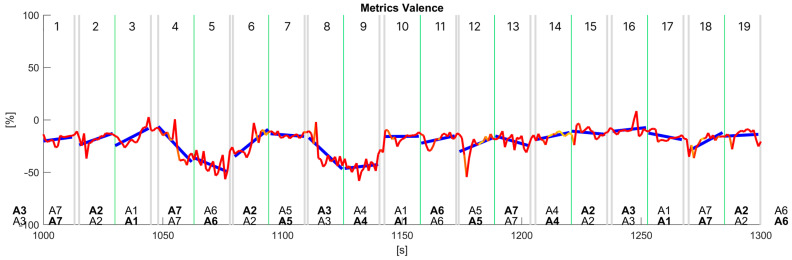
Linear regression analysis of time series data for the valence of ‘juror 4’. Valence data is a sub-selection of raw time series data between the seconds 1000 and 1300 containing complete data about 9 pairwise comparisons. Grey vertical bars separate each pair of audio signals, green vertical lines separate the two signals in each pair.

Further analysis of the collected data involved the application of various statistical techniques, including linear regression, to gain insights into the relationship between the valence signal extracted from facial expressions and the jurors’ choices between each pair of audio signals in the AB comparison with the AB–BA repetition approach. Among the methods employed, utilising a ruleset based on linear regression analysis of the valence data computed at each audio signal via a segmentation analysis approach presented the most promising results, revealing a correlation between the fluctuations in valence and the jurors’ choices in each pairwise comparison. In Figure 8, we show the results of such a linear regression analysis of the time series data for valence data. The text at the bottom of the chart represents the audio signals in each pairwise comparison, where bold means the audio selected by the user to answer the first question, ‘Which is the most annoying sound?’ (top), and the second question, ‘Which one appears to be from a higher-quality car?’ (bottom). When no text is bold, the juror did not choose either the first or second audio. Table 1 shows summaries of the regression analysis, i.e., R2 score, and correlation (⍴) computed using uncorrected (normalised by N) standard deviations for each audio signal.

**Figure 9:**
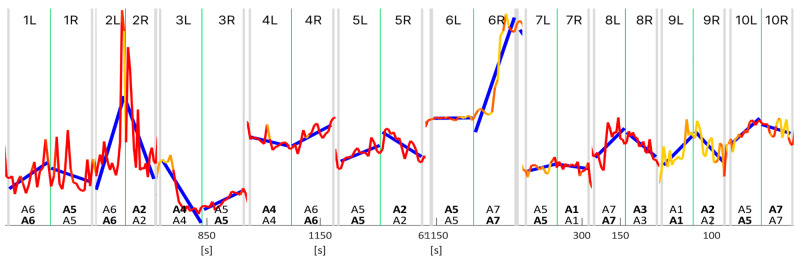
Collection of linear regression analysis of time series data of several pairwise comparisons of different jurors. Grey vertical bars separate each pair of audio signals, green vertical lines separate the two signals in each pair.

The regression analysis was conducted using the trends observed in the valence data to generate a comprehensive list of choices, simulating the jurors’ responses during each pairwise comparison, as shown in Figure 9. Figure 9 is a collection of ten pairwise comparisons cropped from various jurors’ whole time series data. The criterion involved ‘choosing’ the signal based on the relative trend of the regression lines for each signal in every pairwise comparison; i.e., an increase in the valence trend corresponds to the audio signal chosen for the second question. In contrast, a decrease in valence corresponds to the audio signal chosen for the first question. In the cases shown in Figure 9, valence and jurors’ answers showed a good correlation. Table 2 shows summaries of the regression analysis, i.e., R2 score, and correlation (⍴) for each of the numbered pairwise comparisons shown in Figure 9.

However, in other cases, the software could not detect significant variations in valence and engagement, which inevitably led to a reduction in the correlation with the given answers. When jurors express a choice, they do not necessarily feel something on an emotional level, expressing it through a facial expression. In these cases, no significant correlation was found between the valence data derived from facial expressions and the jurors’ responses in the AB comparison methodology. These limitations underscore the complexity of accurately interpreting and analysing emotional data in a controlled jury testing environment. Moreover, an increase in correlation was found where the user appeared to be ‘concentrated’ through the attention recognition tool.

**Figure 10:**
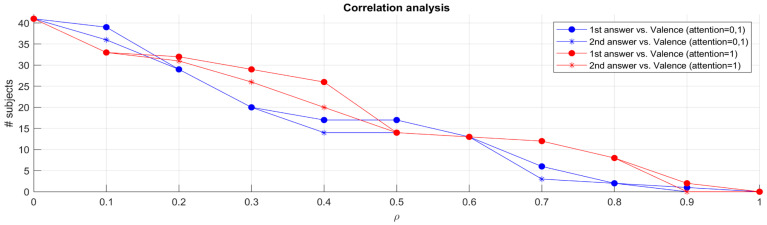
Correlation analysis between valence time series data and answers to questions of all 41 jurors.

By exploiting the above-mentioned criterion of selecting the signal based on the trend of the regression lines, the Spearman correlation between the jurors’ answers to both questions and their valence trends using the corr function was computed in Matlab. Figure 10 shows the correlations computed in such a manner. The lines with dots represent the correlation between the valence time series data and the answers to the first question, and the lines with asterisks represent the correlation between valence data and the second question. The blue lines were computed using all the data independently of the jurors’ attention value (i.e. with an attention value of either 1 or 0). The red lines were computed using only the data where the jurors’ attention value was equal to 1 (i.e., the juror was attentive or looking straight at the camera/monitor).

A higher number of jurors exhibited a higher correlation between their valence and their answers to the questions or different thresholds of the correlation ⍴ (on the X-axis). For instance, if we consider ⍴ = 0.7, we see that six jurors’ data exhibited such a correlation contrary to data from twelve jurors if we select only data where attention equals 1. The increase in the number of jurors exhibiting good correlations, when considering attention data, suggests that heightened attention levels may play a role in the reliability of the answers given by the jurors and, at the same time, in facilitating the recognition and interpretation of emotional cues. The data points to the importance of considering the contextual factors surrounding individual jurors’ engagement levels and attention spans during the testing process, as these factors significantly impact the reliability and accuracy of the emotional data collected.

Although not every juror exhibited trends in their valence data, most correlations were low. The observed correlations emphasize the pivotal role of attentiveness in facilitating a more accurate and nuanced understanding of emotional responses within the jury testing framework.

The identified correlations instill confidence in the potential application of facial expression analysis as a reliable tool for assessing juror engagement but also underscore the potential of leveraging such methodologies to enhance our understanding of people’s behaviour when faced with dichotomous choices.

## 5. Discussion and Conclusions

This study highlights the potential of facial expression recognition tools as a new method for enhancing the accuracy of sound quality jury testing in the products’ design process.

The research’s main objective is to investigate how expression recognition tools could support understanding participants’ levels of valence, engagement, and attentiveness in jury testing. The results highlight the limitations of facial expression recognition software but, at the same time, demonstrate a correlation with the responses given by the jurors in case of recording fluctuations in terms of valence and engagement. Furthermore, the study demonstrates that attention recognition can further increase the statistical correlation between valence and the responses given when the user appears to be attentive.

Expanding from the methodology described for estimating the effective sample size, the decision to include 43 participants in the experimental campaign, exceeding the calculated requirements for both parametric (36) and non-parametric (38) tests derived by G*Power 3 (version 3.1.9.7) analysis, underscores a commitment to robust statistical analysis. This approach not only accommodates the recommended sample sizes determined through G*Power 3 analysis but also provides a buffer to account for potential dropouts or outliers that could affect the study’s power. The inclusion of additional participants ensures that the study maintains its statistical integrity even in the face of unforeseen challenges, enhancing the reliability of the findings. Furthermore, the careful consideration given to the power level and significance level in the experimental design highlights the study’s precision in hypothesis testing. By setting a high power level (1-β) of 0.95 and a stringent significance level (α) of 0.05, the study minimizes the risks of Type II and Type I errors, respectively. Using singular group has been decided by the experimental campaign, and the 7 sounds are representative of the number of measurements. This subtle statistical framework ensures that the study is well equipped to detect true effects, if they exist, while also safeguarding against the identification of false positives.

Regarding the technological limitations of emotion recognition systems from facial expressions, it is essential to cite the results of a recent review study by Barret et al. [49]. They demonstrated that the current ‘emotional facial expression recognition’ technologies are more skilled in detecting facial expressions than emotions. It is well-known that people convey their emotions through facial expressions, such as smiling when happy and frowning when angry. However, this mode of communication can vary depending on cultural differences, specific situations, and individual differences, as only some people display the same facial expressions in response to the same emotions. The interpretation and perception of emotions can vary between individuals, making it challenging to generalise facial expression recognition results to the general population [49]. Another limitation regards the lack of context. Users may have a neutral facial expression when evaluating sound quality, making it difficult to identify any emotional response to the sound. The use of different affective media results in an asymmetry of emotional responses [1]. The same authors experienced this challenge when conducting the tests for this research.

Future studies could improve the reliability and validity of the expression recognition model by using more context-specific datasets integrating other signals, such as biofeedback. Further integration may also be required to improve the attention recognition system currently extracting Boolean values. A fuzzy filter could be added to handle an additional level of uncertainty given by the results of gaze direction and degree of face rotation. Additionally, a wider sample size could help improving the generalizability of the results of the future studies.

However, despite the described limitations, this study has shown the potential of the proposed approach where there is the possibility of having a sufficiently large sample of users or the sounds allow emotional states to be stimulated so that sufficient data emerge to evaluate the jurors’ response in an automated and above all objective manner, without the onset of bias due to distraction.

## Figures and Tables

**Table 1:** R2 score and correlation ⍴ values of the linear regression analysis of time series data for the valence of ‘juror 4’ for each numbered section shown in Figure 8 below.

	R2	⍴
1	0.14	0.37
2	0.31	0.55
3	0.45	0.67
4	0.63	−0.79
5	0.26	−0.51
6	0.62	0.79
7	0.11	−0.33
8	0.51	−0.71
9	0.03	0.19
10	0.00	0.02
11	0.16	0.40
12	0.16	0.39
13	0.24	−0.49
14	0.36	0.60
15	0.03	−0.18
16	0.03	0.18
17	0.21	−0.46
18	0.59	0.77
19	0.01	0.10

**Table 2:** R2 score and correlation ⍴ values of linear regression analysis of time series data for each of the numbered pairwise comparisons shown in Figure 9 below.

	R2 (Left)	⍴ (Left)	R2 (Right)	⍴ (Right)
1	0.37	0.61	0.05	−0.22
2	0.35	0.59	0.40	−0.63
3	0.79	−0.89	0.78	0.88
4	0.23	−0.48	0.52	0.72
5	0.46	0.68	0.50	−0.71
6	0.01	0.09	0.81	0.90
7	0.20	0.45	0.12	−0.34
8	0.35	0.59	0.68	−0.82
9	0.46	0.68	0.45	−0.67
10	0.81	0.90	0.12	−0.35

## Data Availability

Data available on request due to privacy reasons.
